# 

**Published:** 1957-04

**Authors:** 


MEDICAL JOURNAL OF THE SOUTH-WEST

*(Incorporating the Bristol Medico-Chirurgical Journal)*

Established 1883

VOL. 72 (ii) April, 1957 NO. 264

### Contents

| | | Page |
|---|---|---|
| SOME NOTES OF A MEDICAL VISITOR TO THE BRITISH WEST INDIES | *Professor A. V. Neale* | 33 |
| FROM BLUE TO PINK | *Dr. E. G. Bradbeer* | 46 |
| THE PORT HEALTH SERVICE | *Dr. D. T. Richards* | 56 |
| THE SYMPTOMS AND TREATMENT OF ACUTE FOLLICULAR TONSILLITIS | *Dr. S. G. Flavell Matts* | 63 |
| ASTROCYTOMA TREATED BY RADIOTHERAPY: DEATH FROM ACUTE LEUKAEMIA | *Clinical Pathological Conference* | 67 |
| NOTES AND NEWS | | 73 |
| APPOINTMENTS | | 73 |

*PUBLISHED QUARTERLY*

*ANNUAL SUBSCRIPTION 16/- POST FREE*

PUBLISHED UNDER THE AUSPICES OF THE
BRISTOL MEDICO-CHIRURGICAL SOCIETY

### *Editorial Committee*

Dr. J. E. Cates, Department of Medicine, Bristol Royal Infirmary. *Edito*[illegible]

Dr. N. J. Brown, Southmead Hospital, Bristol. *Assistant Editor.*

Dr. J. Macrae, Ham Green Hospital.

Dr. R. C. Wofinden, Central Clinic, Bristol.

Mr. A. E. S. Roberts, Medical Library, University of Bristol. *Secretary.*

### *Local Correspondents*

*Bath* . . Mr. A. D. Bateman, 3 The Circus, Bath.

*Exeter* . . Dr. L. N. Jackson, Mount Jocelyn, Crediton, Devon.

*Gloucester* . Dr. R. F. Jarrett, 9 College Green, Gloucester.

*Plymouth* . Dr. C. R. Croft, Lexden, Hartley Av., Plymouth.

*Taunton* . Dr. M. McAlley, 1 The Crescent, Taunton.

*Torquay* . Dr. A. Robinson Thomas, 20 Devon Square, Newton Abbot, De[illegible]

*Truro* . . Dr. F. D. M. Hocking, St. Andrews, Perranporth, Cornwall.

*Yeovil* . . Mr. J. A. Vere Nicoll, Knapp House, Yeovil, Somerset.

### NOTICE TO CONTRIBUTORS

Original articles are invited, provided they have not been published elsewhere. No[illegible] of forthcoming events, meetings held, degrees conferred, staff appointments, and ot[illegible] local news are welcomed. The editorial committee reserves the right to make liter[illegible] corrections.

Illustrations should always be supplied ready for reproduction. All re-touchings[illegible] other operations required will be charged to the author. Line drawings should be dra[illegible] in Indian ink. We regret that we must ask authors to pay for making blocks, if this[illegible] necessary.

J. W. ARROWSMITH LTD., WINTERSTOKE ROAD,
BRISTOL.

### Notes and News

Dr. Leslie C. Hill delivered the Lumleian lectures on April 2nd and 4th at the Royal College of Physicians. His subject was "Systemic Lupus Erythematosus".

PRIZES

*Crosby Leonard Prize:* R. T. Marcus.

*Francis Dudley Memorial Prize:* D. D. Kennedy.

EXAMINATION RESULTS

*L.R.C.P., M.R.C.S.:* E. E. Bassey, I. E. Doney.

### Appointments

*SOUTH WESTERN REGIONAL HOSPITAL BOARD*

*January to April,* 1957

*BRISTOL*

| *Name* | *Appointment* | *Formerly* |
|---|---|---|
| BAILY, R. A. J., M.B., B.CHIR. (CANTAB.), F.R.C.S. (ENGLAND). | Consultant Orthopaedic Surgeon to the Weston-super-Mare Group of Hospitals. | Hon. Clinical Assistant in Orthopaedic Surgery at the Royal London Homoeopathic Hospital. |
| LANCASTER, N. P., M.D. (MANCHESTER), D.P.M. (LONDON). | Consultant Psychiatrist, Bristol Mental Hospitals. | Assistant Psychiatrist at Deva Hospital, Chester. |
| BORG, F. X., M.D. (MALTA), D.A. (ENGLAND). | Anaesthetic Registrar, Southmead Hospital. | Anaesthetic Registrar, Bedford Group of Hospitals. |
| HILL, B. W., M.B., CH.B. (BRISTOL). | Clinical Assistant in Dermatology, Cossham Hospital. | In general practice in Bristol. |
| WETHERED, R. R., B.A., B.M., B.CH. (OZON.). | Clinical Assistant in Dermatology, Southmead Hospital. | In general practice in Bristol. |
| BANERJEE, A. K., M.B., B.S. (CALCUTTA), F.R.C.S. (ENG.) AND (EDIN.). | Surgical Registrar, Southmead Hospital. | Royal Devon and Exeter Hospital, Exeter. |
| TALBOT, C. H., M.A., M.B., B.CHIR. (CAMB.), F.R.C.S. (ENG.). | Senior Surgical Registrar, Southmead Hospital (joint appointment with United Bristol Hospitals). | Surgical Registrar, United Sheffield Hospitals. |
| WATKINS, E. S., B.SC. (LIVERPOOL), M.D. (LIVERPOOL). | Surgical Registrar, Weston-super-Mare General Hospital. | Winford Orthopaedic Hospital. |
| RUSSELL, MISS P. M., M.D. (LIVERPOOL), D.(OBST.) R.C.O.G., D.C.H. | Registrar in Obstetrics and Gynaecology, Southmead Hospital. | Resident Gynaecological Pathologist, Jessop Hospital for Women, Sheffield. |
| STATHER-DUNNE, M. T., M.B., B.S. (LONDON), D.P.M. (PART 1). | Registrar, Bristol Mental Hospitals. | S.O.H., Bristol Mental Hospitals. |

*NORTH GLOUCESTERSHIRE*

| *Name* | *Appointment* | *Formerly* |
|---|---|---|
| HALLORAN, MISS A., M.B., B.S. (SYDNEY), F.R.C.S. (EDIN.). | Surgical Registrar, Gloucestershire Royal Hospital, Gloucester. | — |

### *NORTH GLOUCESTERSHIRE*—continued.

| *Name* | *Appointment* | *Formerly* |
|---|---|---|
| FOY, B. N., M.B., B.S. (LONDON). | Registrar in Obstetrics and Gynaecology, Gloucestershire Royal Hospital, Gloucester. | Locum Registrar in Obstetrics and Gynaecology, St. Alfege's Hospital, Greenwich. |

### *BATH*

| *Name* | *Appointment* | *Formerly* |
|---|---|---|
| COSH, J. A., M.A. (CAMBRIDGE), M.D. (CAMBRIDGE), M.R.C.P. (LONDON). | Consultant Physician to the Bath Clinical Area. | Lecturer in Medicine, University of Bristol, Hon. Asst. Physician to United Bristol Hospitals, Hon. Consultant Physician to Bristol Clinical Area. |
| MOORE, E. W., M.B., CH.B. (BRISTOL), D.P.H. (BRISTOL) M.R.C.P. (EDINBURGH), D.M.R.D. (CONJOINT BOARD). | Consultant Radiologist to the Bath Clinical Area. | Senior Registrar in Radiodiagnosis, University College Hospital, London. |
| CHANDRASEKARAN, M.S.I., M.B., B.S. (MADRAS), D.L.O. (PART 1). | E.N.T. Registrar, Bath Group of Hospitals. | S.H.O. in E.N.T. Surgery, Eye, Ear and Throat Hospital, Shrewsbury. |

### *SOUTH SOMERSET*

| *Name* | *Appointment* | *Formerly* |
|---|---|---|
| BATHELL, M. F., M.A., M.D. (CAMBRIDGE), D.P.M. | Consultant Psychiatrist and Deputy Medical Superintendent, Tone Vale Hospital, nr. Taunton. | Consultant Psychiatrist, Cheadle Royal Hospital, Cheshire. |
| KNAPPE, S. S., M.D. (WARSAW). | Deputy Medical Superintendent, Sandhill Park Hospital, Bishop's Lydeard. | Assistant Psychiatrist, Hensol Castle, Pontyclun, Glam. |

### *DEVON AND CORNWALL*

| *Name* | *Appointment* | *Formerly* |
|---|---|---|
| REECE, M. W., M.B., B.S. (LONDON), F.R.C.S. (ENGLAND). | Senior Surgical Registrar, Royal Devon and Exeter Hospital, Exeter (joint appointment with the United Bristol Hospitals). | Surgical Registrar, United Sheffield Hospitals. |
| COX, M. G., M.A., M.B., B.CHIR., F.R.C.S. (ENGLAND). | E.N.T. Registrar, South Devon and East Cornwall Hospital, Plymouth. | S.H.O., E.N.T. Department, Salisbury General Hospital. |
| DUKE, R. F. N., B.M., B.CH. (OXFORD). | Orthopaedic Registrar, Torbay Hospital, Torquay. | S.H.O. in Orthopaedics, St. James' Hospital, Balham. |

